# Score to scan: Is there a link between Glasgow Coma Scale score and CT neuroimaging findings in trauma?

**DOI:** 10.4102/sajr.v30i1.3304

**Published:** 2026-02-20

**Authors:** Kavishka Sewnarain, Shalendra K. Misser, Jaynund Maharajh, S. Sameer Nadvi

**Affiliations:** 1 Department of Radiology, School of Medicine, College of Health Sciences, University of KwaZulu-Natal, Durban, South Africa; 2 Department of Radiology, College of Health Sciences, Inkosi Albert Luthuli Central Hospital, Durban, South Africa; 3 Department of Radiology, Faculty of Health Sciences, Lake Smit and Partners, Durban, South Africa; 4 Department of Radiology, Faculty of Health Sciences, King Edward VIII Hospital/Victoria Mxenge Hospital, Durban, South Africa; 5 Department of Neurosurgery, School of Medicine, College of Health Sciences, University of KwaZulu-Natal, Durban, South Africa

**Keywords:** computed tomography, extradural haemorrhage, Glasgow Coma Scale, head injury, neurosurgical intervention, subarachnoid haemorrhage, subdural haemorrhage, traumatic brain injury

## Abstract

**Background:**

Many South African peripheral medical centres lack direct access to CT scans or neurosurgery. The Glasgow Coma Scale (GCS), used with or without other findings, remains widely utilised in traumatic brain injury (TBI) assessments with lack of standardisation between centres. There is limited data from South Africa (SA) correlating GCS scores to CT imaging in TBI.

**Objectives:**

This study aimed to assess CT findings at various GCS levels to determine whether GCS was a reliable indicator for imaging and referral.

**Method:**

A retrospective review of 385 patients categorised with mild, moderate or severe TBI was performed. The initial non-sedated post-resuscitation GCS score and initial CT brain findings were compared using the chi-square and Fisher’s exact tests.

**Results:**

Increased intracranial pressure and subdural haemorrhage occurred in 41.7% and 53.7% of patients with GCS 9–12, respectively, and 30.5% and 41.4% of patients with GCS 13–15, respectively. The highest incidence of depressed skull fractures (51.3%; 95% confidence interval [CI], 43.2–59.3%; *p* < 0.001) and pneumocephalus (25.6%; 95% CI, 42.2–56.4; *p* < 0.001) were reported in the CGS 13–15 category. Neurosurgical intervention was required in 83.2% and 73.0% of patients with GCS scores of 9–12 and 13–15, respectively.

**Conclusion:**

The severe category of GCS predicts imaging and neurosurgery requirements while the mild to moderate categories underpredict the need for patient referral.

**Contribution:**

This study provides rationale for the development of a local, standardised assessment tool to guide referral of TBI patients for imaging in resource-limited settings.

## Introduction

The incidence of trauma in South Africa (SA) is almost the highest globally, with traumatic brain injury (TBI) being the most common cause for high morbidity and mortality.^1^ This highlights the strain on ambulance services and tertiary trauma centres in a resource-constrained country.^2^

With limited access to neurosurgical care, TBI management is predominantly performed by generalists, paramedical and referring staff.^3^ Although other scoring systems exist, the Glasgow Coma Scale (GCS) remains popular because of its simplicity and widespread application.^4^ The GCS, described in 1974 by Teasdale and Jennet,^5,6^ allows healthcare workers to quickly evaluate the severity of TBI.^6^ A GCS score of 13–15 is characterised as mild, 9–12 as moderate and 3–8 as severe TBI.^7,8^ However, a GCS score of 15 does not necessarily exclude TBI.^9^ In addition to the adult GCS, a modified paediatric GCS that considers age-appropriate developmental variations of cognitive, verbal and motor skills was developed for preverbal children.^10^ The accuracy of GCS assessments can be reduced with sedation, paralysing agents, presence of facial injuries and intubation.^7^

The Canadian CT Head Rule used for mild TBI categorises GCS scores of < 15 at 2 h following injury, multiple episodes of vomiting, age > 65 years, open or depressed fractures or features of base of skull fractures as high risk for neurosurgery, while > 30 min retrograde amnesia and a dangerous mechanism of injury are categorised as medium risk for abnormal CT findings.^11^ The New Orleans criteria consider the presence of headache, vomiting, persisting anterograde amnesia, seizures, visualised trauma above the clavicle, age > 60 years or alcohol or drug intoxication in a patient with a GCS score of 15 as requiring imaging.^12^ The inherent differences between scoring systems and their varied utilisation between peripheral centres result in confusion and lack of standardised management.

The clinical decision tools for TBI imaging have been predominantly created in developed countries with different financial constraints, healthcare staffing and trauma burdens compared to developing countries such as SA.^2^ In 2018, SA had five CT scanners per million people compared with 41 and 101 scanners per million people in the United States of America and Japan, respectively.^13^ In SA, where the ratio of patients to CT scanners is among the highest globally,^13^ local guidelines are required. Because of the lack of standardised national guidelines, several locally developed guidelines are utilised with poor compliance.^3^

Public sector hospitals, which are far removed from tertiary institutes, have limited access to CT scanners resulting in a large number of patients with TBI requiring transfer to a tertiary or quaternary hospital.^14^ The findings of this study are aimed to help paramedics and staff in peripheral and rural hospitals who face resource constraints and lack direct access to imaging and neurosurgery. To date, little research has been conducted locally to assess the predictive utility of the GCS for CT imaging abnormalities and neurosurgical referral. Establishing the association between GCS scores and CT findings in the South African population would be the first step in the development of an evidence-based TBI assessment tool that could be tailored to local healthcare models, resources and epidemiology.

This study aimed to compare the GCS scores of TBI patients at initial presentation to CT findings. By analysing the CT findings in mild, moderate and severe GCS categories, it could be determined if initial GCS values can reliably predict intracranial trauma on CT and assist in the development of evidence-based triage protocols and indications for CT imaging.

## Research methods and design

### Study design, setting and population

A retrospective review of 385 adult and paediatric patients who presented with closed TBI to Inkosi Albert Luthuli Central Hospital (IALCH), Durban, SA was conducted from January 2018 to January 2020. The IALCH, a quaternary hospital, receives patients from KwaZulu-Natal and parts of the Eastern Cape provinces. All patients who required a CT scan for closed TBI during the study period were included. Patients with open head injuries and other medical causes that could account for a low GCS score were excluded. The study focused on post-resuscitation GCS scores of included patients, prior to sedation and initial unenhanced CT brain imaging. Patients were scanned on either the Siemens Definition flash or the Siemens Definition AS+.

For this study, a GCS score of 13–15 was categorised as mild, 9–12 as moderate and 3–8 as severe (Table 1).^7^ Patients who were categorised as not specified or unknown in this study were not included in the statistical analysis. A modified paediatric GCS was utilised in preverbal patients (Table 1).^10^

**TABLE 1:** Numbers and percentages of patients presenting within the various categories of the Glasgow Coma Scale^7^ and paediatric Glasgow Coma Scale.^10^

Response	Glasgow Coma Scale score	*n*	%
**Eye opening**	-	385	-
Opens eyes spontaneously	4	229	64.2
Paediatric: Spontaneous eye opening	-	1	-
Opens eyes to verbal command	3	48	13.4
Paediatric: Eye opening to voice	-	0	-
Opens eyes to pain	2	38	10.6
Paediatric: Eye opening to pain	-	0	-
Does not open eyes to any stimulation	1	42	11.7
Paediatric: No eye opening	-	0	-
Not specified†	-	27	-
Paediatric: Not specified	-	0	-
**Motor response**	-	385	-
Follows commands	6	205	57.5
Paediatric: Spontaneous movement	-	1	-
Localises painful stimuli	5	104	29.0
Paediatric: Withdrawal to touch	-	0	-
Flexion or withdrawal to pain	4	29	8.1
Paediatric: Withdrawal to pain	-	0	-
Abnormal flexion in response to pain	3	6	1.7
Paediatric: Abnormal flexure posturing	-	0	-
Extension in response to pain	2	6	1.7
Paediatric: Abnormal extension posturing	-	0	-
No response to stimuli	1	7	2.0
Paediatric: No response	-	0	-
Not Specified‡	-	27	-
Paediatric: Not specified	-	-	-
**Verbal response**	-	385	-
Orientated to time place and person	5	89	25.1
Paediatric: Coos or babbles	-	1	-
Confused	4	102	28.5
Paediatric: Irritable or crying	-	0	-
Inappropriate response	3	42	11.7
Paediatric: Cries in response to pain	-	0	-
Incomprehensible speech	2	58	16.2
Paediatric: Moans	-	0	-
No response	1	52	14.5
Paediatric: Absence of verbal response	-	0	-
Intubated	T	14	3.9
Not Specified§	-	27	-
Paediatric: Not specified	-	-	-

Note: Please see full reference list of this article, Sewnarain K, Misser SK, Maharajh J, Nadvi SS. Score to scan: Is there a link between Glasgow Coma Scale score and CT neuroimaging findings in trauma? S Afr J Rad. 2026;30(1), a3304. https://doi.org/10.4102/sajr.v30i1.3304, for more information.

† , Patients in whom the individual eye opening information was not documented;

§ , Patients in whom the the individual verbal response information was not documented;

‡ , The individual component score for GCS score was not provided for 27 patients.

The sample size of 385 was calculated as follows: by assuming that the prevalence of TBI in SA is 50%, based on historic data,^15^ with a 95% confidence level and a precision level of 5% (Equation 1):


$$\begin{array}{l} n=\frac{z^{2}(p)(1-p)}{\varepsilon^{2}}\\ n=\frac{1.96^{2}(0.5)(1-0.5)}{0.05^{2}}\\ n=385 \end{array}$$ [Eqn 1]

where *n* is the required sample size, *Z* is the z-score corresponding to the desired confidence level, *p* is the estimated proportion of an attribute present in the population, and ε^2^ is the desired margin of error squared.

### Data collection and analysis

Consecutive sampling was used. Data were collected from the hospital online records using a data recording form. All patient identifying information was removed.

The initial, unenhanced CT brain scans of all included patients were blinded and independently reviewed by the principal investigator, a qualified neuroradiologist. These findings were then compared to the formal reports. Where any discrepancies arose, a second neuroradiologist, blinded to both the reports and the principal investigator’s findings, reviewed the findings as an adjudicator. Any disagreements were discussed at a consensus meeting. Images were reviewed using the brain, soft tissue, lung and bone window presets.

The following variables were identified and analysed: GCS scores (GCS score categories of 3–6, 9–12 and 13–15); CT brain findings (midline shift as ≤ 5 mm or > 5 mm, extradural haemorrhage [EDH], subdural haemorrhage [SDH], subarachnoid haemorrhage [SAH], intraparenchymal haemorrhages, intraventricular haemorrhage [IVH], hydrocephalus, diffuse axonal injury [DAI], intracranial herniations [subfalcine, uncal, ascending transtentorial, descending transtentorial, tonsillar, ascending transalar, descending transalar and extracranial], cerebral atrophy, subgaleal haematoma, scalp foreign body, haemosinus, effacement of sulci, effacement of cisterns, cerebral oedema, vasogenic oedema, pneumocephalus, tension pneumocephalus, intra-axial foreign body, generalised raised intracranial pressure [ICP], focal raised ICP, skull fracture, linear fracture, comminuted fracture, depressed fracture, none of these parameters); neurosurgical intervention required; outcome (down referred, discharged, demised, unknown) and length of admission (< 10 days, ≥ 10 days, unknown).

Rotterdam CT scoring was used to categorise data. However, because it is predominantly used for assessment of mortality risk based on the basal cistern, midline shift, epidural lesion and presence of IVH or SAH,^16^ additional CT findings were assessed for and documented, as this study focuses on TBI severity to expedite referral to radiology and neurosurgery.

Complete case analysis and no additional data, other than what is discussed above, were used in the statistical analysis plan. All data were captured into Statistical Packages for the Social Sciences version 25 for analysis. A *p*-value < 0.05 was deemed statistically significant. The study was exploratory. The results were not adjusted for multiplicity, and results were only presented descriptively. Measures of central tendency (i.e. mean and median) were used to summarise continuous variables while frequency distribution tables and graphs were used to present categorical data. The chi-square and Fisher’s exact test were used to assess for any statistical associations between categorical variables.

### Ethical considerations

Ethical approval was obtained from the Biomedical Research Ethics Committee at the University of KwaZulu-Natal, Durban, SA on 06 December 2019 (approval number: BE 430/19). Additionally, permission to conduct the study was also obtained from the KwaZulu-Natal Department of Health, Department of Neurosurgery and medical manager of IALCH. All research performed in this study that involved human participants was in accordance with the ethical standards of the institutional and National Research Committee in line with the 1964 Helsinki Declaration and its later amendments.

## Results

The majority of patients were 17–64 years old (*n* = 353; 91.7%), followed by ≤ 16 years old (*n* = 24; 6.2%), and ≥ 65 years old (*n* = 8; 2.1%). In the ≤ 16 years old category, the youngest patient was 2 days old, while the rest were all ≥ 10 years old.

**FIGURE 1:**
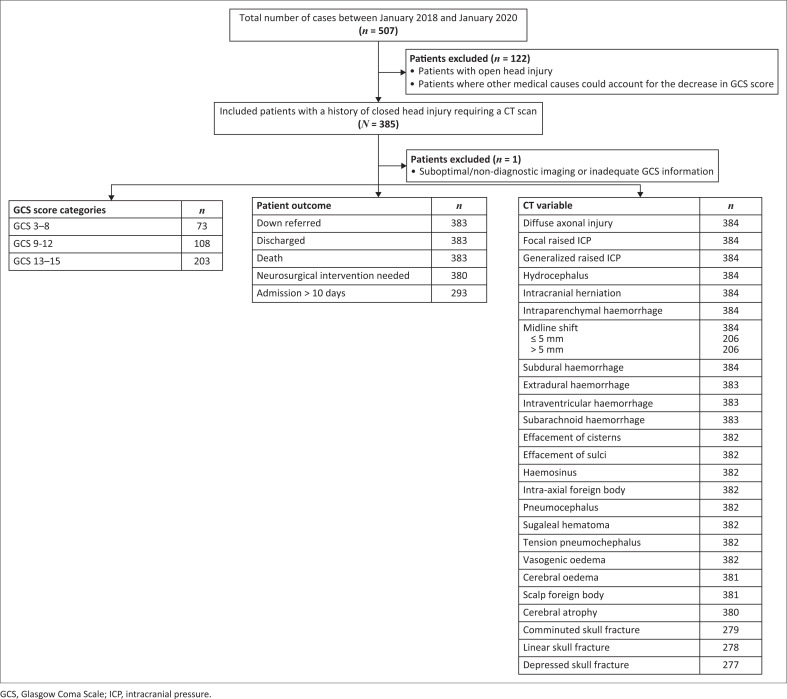
Patient disposition and baseline study characteristics.

The paediatric GCS scale was utilised for the single preverbal patient who obtained a score of 15. A GCS score of 13–15, 9–12 and 3–8 was documented for 203 (52.9%), 108 (28.1%) and 73 (19.0%) patients, respectively (Figure 1). The GCS was unknown in one patient. The majority of patients in the eye-opening and motor response category had the highest score in their respective GCS subcategory (64.2% and 57.5%, respectively), whereas most patients in the verbal response category were reported as confused (28.5%, Table 1).

### Glasgow Coma Scale score 3–8

**FIGURE 2:**
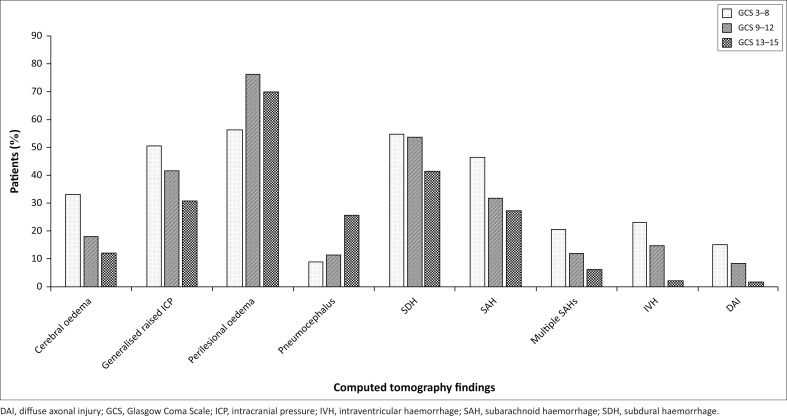
Multiple CT findings per Glasgow Coma Scale category.

An initial GCS score of 3–8 was associated with global cerebral oedema in 32.9% of patients (*n* = 24; 95% confidence interval [CI], 22.3–44.9; *p* < 0.001), generalised raised ICP in 50.7% of patients (*n* = 37; 95% CI, 38.7–62.6; *p* = 0.005) and SDH in 54.8% of patients (*n* = 40; 95% CI, 42.7–66.5; *p* = 0.043). Compared with other GCS score categories, there was a higher incidence of SAH (*n* = 34; 46.6%; 95% CI, 42.7–66.5; *p* = 0.009), IVH (*n* = 17; 23.3%; 95% CI, 14.2–34.7; *p* < 0.001) and DAI (*n* = 11; 15.1%; 95% CI, 7.8–25.4; *p* < 0.001) in the GCS 3–8 category (Figure 2).

### Glasgow Coma Scale score 9–12

Perilesional oedema was documented in 76.4% (*n* = 81) of patients in the GCS 9–12 category (95% CI, 67.2–84.1; *p* = 0.015). Generalised raised ICP, SDH, and SAH were documented in 41.7% (*n* = 45), 53.7% (*n* = 58) and 31.8% (*n* = 34) of patients, respectively. Global cerebral oedema was documented in 17.9% (*n* = 19) of patients (Figure 2).

### Glasgow Coma Scale score 13–15

Pneumocephalus occurred more frequently in the GCS 13–15 category (*n* = 52; 25.6%; 95% CI, 42.2–56.4; *p* < 0.001) compared with the other categories. Perilesional oedema (*n* = 142; 70%) had the second highest incidence in this category. Generalised raised ICP, SDH and SAH were documented in 30.5% (*n* = 62), 41.4% (*n* = 84) and 27.1% (*n* = 55) of patients, respectively. Moreover, cerebral oedema occurred in 11.9% (*n* = 24) of patients in this category (Figure 2).

### Fractures and Glasgow Coma Scale

Skull fractures had the highest incidence in the GCS 13–15 category at 77.8% (*n* = 158, 95% CI, 71.5–83.4; *p* = 0.024), compared with 70.4% (*n* = 76) in the GCS 9–12 and 61.6% in the GCS 3–8 category.

**FIGURE 3:**
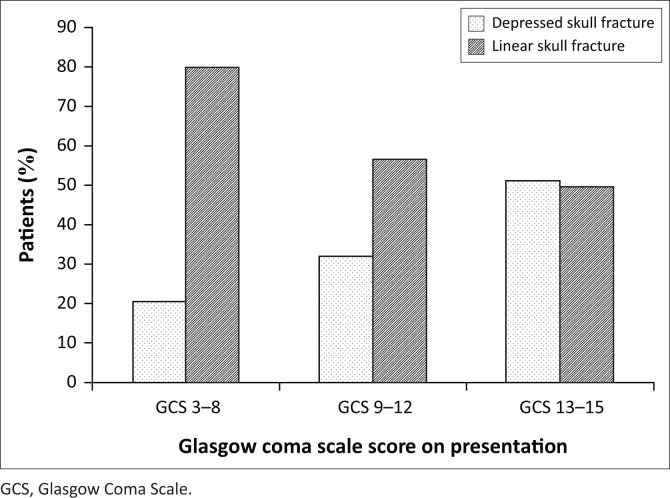
Types of skull fractures at various Glasgow Coma Scale categories.

The incidence of linear fractures was higher in patients in the GCS 3–8 category (*n* = 36; 80.0%; 95% CI, 65.4–90.4; *p* = 0.001), whereas depressed fractures had a higher incidence in the GCS 13–15 category (*n* = 81; 51.3%; 95% CI, 43.2–59.3; *p* < 0.001; Figure 3). Among all patients with a skull fracture, 65.6% were associated with an extra axial haemorrhage and 68.1% with intraparenchymal haemorrhage.

### Outcome

**FIGURE 4:**
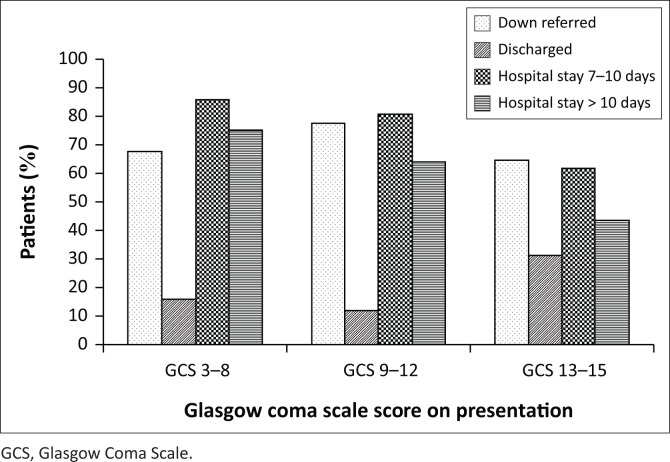
Outcomes at various Glasgow Coma Scale categories.

The majority of patients (*n* = 268; 69.6%) were down referred, 23.1% (*n* = 89) were discharged and 7.0% (*n* = 27) demised. Most patients who were down referred had GCS scores of 9–12 (*n* = 84; 78.5%; 95% CI, 69.5–85.9; *p* < 0.001; Figure 4), most discharged patients had GCS scores of 13–15 (*n* = 64; 31.5%; 95% CI, 25.2–38.4; *p* < 0.001) and the majority who died were from the GCS 3–8 category (*n* = 11; 15.1%; 95% CI, 7.8–25.4; *p* < 0.001). Deaths in the GCS 9–12 category amounted to 9.3% (*n* = 10) and in the GCS 13–15 category to 3.0% (*n* = 6). Two patients were excluded because of missing data.

Based on the Rotterdam CT classification,^16^ 5.4% of patients in the GCS 13–15 category, 5.5% in the GCS 3–8 category and 6.5% in the GCS 9–12 category obtained a score of 5. Moreover, 2.0% of patients in the GCS 13–15 category, 1.9% in the 9–12 category and 9.6% in the GCS 3–8 category scored 6 (Table 2).

**TABLE 2:** Comparison of Glasgow Coma Scale score categories to Rotterdam CT scores.

GCS scorecategory†	Rotterdam CT score‡											
	1		2		3		4		5		6	
	*n*	%	*n*	%	*n*	%	*n*	%	*n*	%	*n*	%
GCS 13–15 (*n* = 203)	29	14.3	90	44.3	53	26.1	16	7.9	11	5.4	4	2.0
GCS 9–12 (*n* = 107)	10	9.0	32	30.0	36	33.6	20	18.7	7	6.5	2	1.9
GCS 3–8 (*n* = 73)	7	9.6	19	26.0	20	27.4	16	21.9	4	5.5	7	9.6

Note: Please see full reference list of this article, Sewnarain K, Misser SK, Maharajh J, Nadvi SS. Score to scan: Is there a link between Glasgow Coma Scale score and CT neuroimaging findings in trauma? S Afr J Rad. 2026;30(1), a3304. https://doi.org/10.4102/sajr.v30i1.3304, for more information.

GCS, Glasgow coma score.

† , Two patients excluded because of missing data;

‡ , Basal cisterns: normal = 0, compressed = 1, absent = 2. Midline shift: absent or ≤ 5 mm = 0, > 5 mm = 1. Epidural mass: present = 0, absent = 1. Intraventricular blood or traumatic subarachnoid haemorrhage: absent = 0, present = 1. The individual scores are added and 1 added to the total. The mortality associated with a score of 1 was 0%, with 2 was 6.8%, with 3 was 16%, with 4 was 26%, with 5 was 53% and with 6 was 61%.^16^

Neurosurgical intervention was required in 298 of 385 patients with TBI (77.4%), with 86.3% (*n* = 63; 95% CI, 76.3–93.2; *p* = 0.023) with a GCS between 3 and 8 requiring neurosurgical intervention. In the GCS 9–12 and 13–15 categories, neurosurgical intervention was required in 83.2% (*n* = 89) and 73.0% (*n* = 146) of patients, respectively. Five patients were excluded because of missing data.

Patients in the GCS 3–8 category had longer hospital admissions with 75.9% (*n* = 41; 95% CI, 62.4–86.5, *p* < 0.001) admitted for > 10 days (Figure 4). In the GCS 9–12 and 13–15 categories, 64.9% and 43.8% of patients were admitted for > 10 days, respectively. When considering presenting symptoms, signs and GCS, 40.5% of patients improved, 34.3% remained in status quo and 15.6% worsened on down referral or discharge.

## Discussion

This study provides insight into the CT imaging profile of various GCS score categories. Mild TBI was most prevalent at 52.9%, and although lower than the previously reported incidence of 87.5% in SA,^17^ it still represents a large proportion of patients. The study found that GCS underestimates severity, imaging and neurosurgery requirements in mild and moderate TBI, which comprised 81.0% of patients collectively. Therefore, closer attention is required in patients in these categories to ensure appropriate assessment with standardised protocols and timeous referral for imaging or neurosurgical evaluation.

Globally, approximately 85% of the population live in low- and middle-income countries (LMIC), which also have the highest incidence rates of TBI.^18^ Although TBI incidence in LMIC may still be underestimated, its incidence is approximately three times higher than in high-income countries.^19^ In LMIC, such as SA, neuroimaging and neurosurgical facilities are not readily available in many parts of the country. It is often decided by assessment at the scene whether patients would require specialist care, which may require longer transport times.^20^

Although fewer patients in the GCS 9–12 and GCS 13–15 categories demised compared with the GCS 3–8 category, the difference was only 2.7% higher in the GCS 3–8 category compared with the other categories combined. In the Rotterdam CT score classification, patients scoring 5 and 6 demonstrated a 53% and 61% mortality rate, respectively.^16^ As anticipated, the GCS 3–8 category had the highest percentage of patients in the Rotterdam CT 5 and 6 categories combined (15.1%), followed by the GCS 9–12 category (8.4%) and the GCS 13–15 category (7.4%), highlighting the proportion at risk in the mild and moderate GCS categories. A study from Ethiopia reported a cumulative survival rate of 36.9% in patients with severe TBI and 46.6% and 75.0% in moderate and mild TBI, respectively.^21^ Although the difference between the GCS categories is comparatively larger than that in the current study, the percentage of patients who demised in the mild and moderate categories is still significant and should not be overlooked.

Although the GCS 3–8 category in this study had the highest percentage of patients requiring neurosurgical intervention (86.3%), it was only 3.1% higher than the moderate category. The rates of neurosurgical intervention in the mild TBI category vary between 0.7% – 8.7%^22^ and 20.2%.^23^ These rates are lower than the 73.0% reported in this study. A possible explanation for the higher rate of neurosurgical intervention is that IALCH is a quaternary referral centre.

**FIGURE 5:**
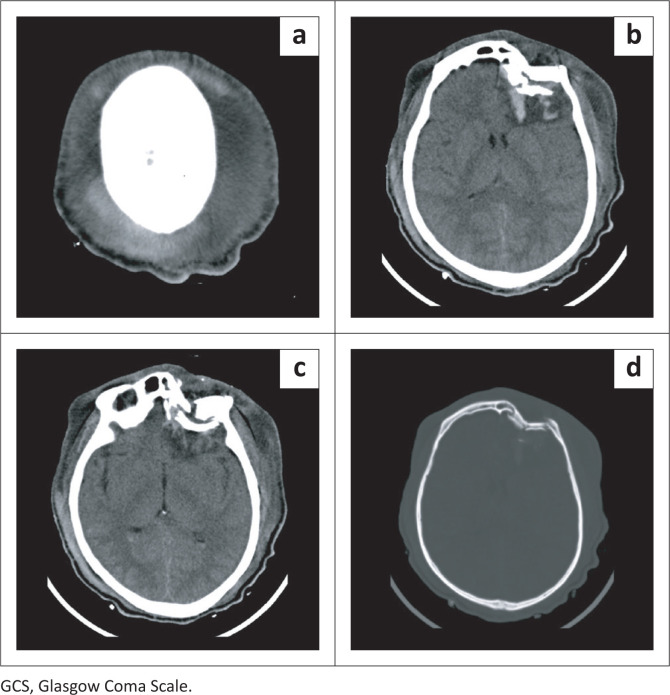
Twenty six-year-old male post fall from a moving vehicle with an initial Glasgow Coma Scale score of 7 received an axial unenhanced CT brain scan on brain (a, b, c) and bone window setting (d). CT imaging indicates extensive subgaleal soft tissue swelling (a), depressed comminuted left frontal skull fracture (b, c, d) with underlying intraparenchymal haemorrhages and perilesional oedema (b, c), left frontal subarachnoid haemorrhage (b, c) and intraventricular haemorrhage in the occipital horns of the lateral ventricles bilaterally (c).

Similar to Morgado et al. who found that a low GCS score was associated with a greater number of CT findings,^24^ this study confirmed that the GCS 3–8 category correctly identified severe TBI with the possibility of severe intracranial pathology, demonstrating a significantly higher incidence of cerebral oedema, generalised raised ICP, IVH (Figure 5) and DAI. In this study, patients with severe TBI had a higher incidence of SDH and SAH, which is consistent with previous findings reported by Wardlaw *et al*.^25^

**FIGURE 6:**
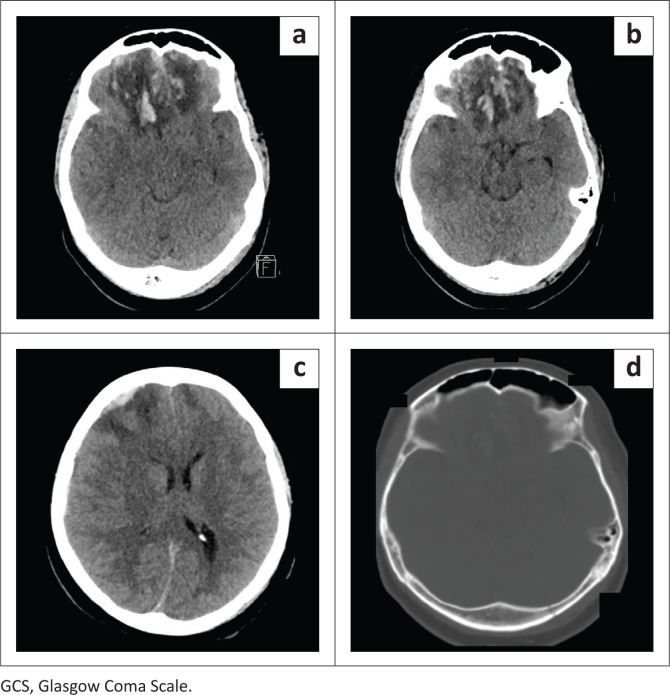
Thirty three-year-old female patient with history of fall from a moving vehicle presented with a Glasgow Coma Scale score of 13. Axial CT brain images on brain (a, b, c) and bone window setting (d) demonstrating bifrontal intraparenchymal and subarachnoid haemorrhages, perilesional oedema, frontal interhemispheric subdural haemorrhages (a), effacement of the basal cisterns (b), right frontal epidural haemorrhage, midline shift (c) and left occipital linear fracture (d).

**FIGURE 7:**
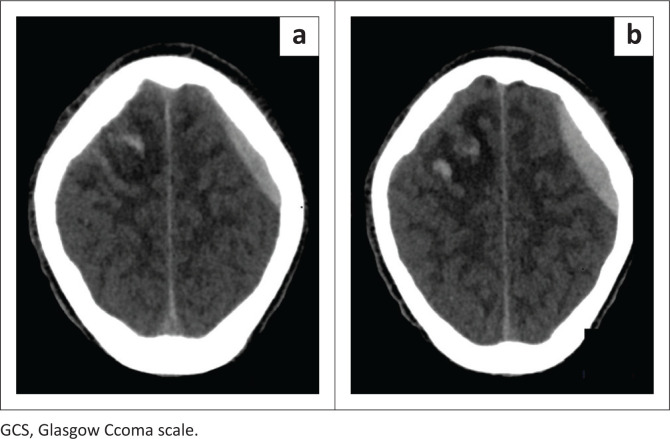
Seventeen-year-old male, post assault with a blunt object with an initial Glasgow Coma Scale score of 15 on presentation. Axial CT brain unenhanced on brain setting (a, b) demonstrates right frontal subarachnoid haemorrhage (a), interhemispheric subdural haemorrhage (a, b), right frontal intraparenchymal haemorrhage (b), perilesional oedema (a, b), left frontal and parietal extra dural haemorrhage (a, b).

Adequately selecting patients with mild TBI for CT imaging allows for prompt identification of those requiring surgery or hospitalisation.^20^ Although the incidence of generalised raised ICP in this study was numerically the highest in the GCS 3–8 category (*n* = 37; 50.7%, Figure 2), the number of patients in the combined moderate and mild categories (*n* = 107) was higher when compared with the severe category. Similarly, although the incidence of SDH and SAH was higher in the GCS 3–8 category (54.8% and 46.6%, respectively), the incidence in patients with moderate or mild TBI was not markedly lower (Figure 2) and should not be overlooked. Figure 6 and Figure 7 demonstrate the findings in patients with a GCS score of 13 and 15, respectively. A study from Malaysia that considered SDH, EDH, SAH, IVH, intraparenchymal haemorrhage, contusion, pneumocephalus and depressed skull fractures as significant CT findings, demonstrated significant findings in 53.2% of patients in the GCS 13–15 category with 75.0% of patients with a GCS score of 13 or 14 demonstrating significant CT findings.^23^ In this study, although perilesional oedema had the highest incidence in the GCS 9–12 category, it was present in 70% of patients in the GCS 13–15 category. This reiterates the need for a standardised assessment for patients, including in mild TBI, to ensure appropriate management.

Fractures with displacement of bone more than the width of the adjacent skull are defined as depressed fractures.^26,27^ These had a higher incidence in the GCS 13–15 category (51.3%; *p* < 0.001) in this study. The presence of skull fractures can alert to underlying extra or intra-axial haemorrhages, with prior studies reporting EDH in 35% of those with linear skull fractures.^26^ Skull fractures were associated with extra-axial haemorrhages in 65.6% and intraparenchymal haemorrhages in 68.1% of patients in this study. Therefore, despite a GCS score of 13–15, the presence of skull fractures should raise concern of intracranial injury. IALCH neurosurgery guidelines state that patients with a decreased level of consciousness associated with depressed skull fractures or features of base of skull fractures require urgent CT scanning. However, patients with a GCS score of 15, along with a closed linear skull fracture on radiograph and no focal deficits, are eligible for elective CT scanning. Prior research shows varied results regarding CT findings in mild TBI, with some documenting normal scans in 93.2% of patients presenting with a GCS score of 15,^28^ while positive CT findings were recorded in 29.2% of patients in another study.^29^ In contrast to this, none of the patients in the GCS 13–15 category in this study had a normal CT scan. This could be accounted for by the centre being a referral hospital. As a referral hospital there will naturally be a higher percentage of positive CT scans of mild and moderate TBI as patients may have been referred based on other factors such as mechanism of injury. This is of particular significance in comparison with other hospitals that accept all patients, which will have a higher number of negative CT scans. Future multicentre prospective studies at walk in hospitals may be of significance.

A study from Canada reported longer hospital admission in the GCS 3–8 category.^30^ In this study, the highest percentage of patients admitted for > 10 days (75.9%) were from the GCS 3–8 category. This plays a role in planning for the utilisation of resources. Previous research from SA has demonstrated a lower rate (28.2% – 60.0%) of favourable outcomes in patients with severe TBI when compared with those from high-income countries.^31^ A shorter time interval between injury and admission into neurosurgery and mild TBI on admission are significant predictors of a favourable outcome.^1^ The incidence of patients who die prior to reaching the hospital in LMIC is more than double of that in high-income countries^32^ with approximately 50.0% dying within the first two hours following injury.^32^ Therefore, prompt patient assessment, stabilisation and appropriate referral are imperative.

### Limitations

Study limitations include that the mechanism of injury and other signs of head injury, including focal neurology, were not included in the assessment of TBI severity. Moreover, the timeframe between injury to clinical assessment was not documented. Future studies incorporating mechanism of injury, clinical signs and focal neurology will assist in categorising TBI severity. Prospective studies will assist in determining otherwise poorly documented data, such as timeframe between injury and clinical assessment. Although performed at a tertiary institute, the study was aimed at peripheral institutes but could not be performed in peripheral centres because of the absence of imaging and neurosurgical services.

This study was based at IALCH, a centre to which many peripheral emergency departments refer. Many patients with TBI would not have access to IALCH or CT imaging as not all patients with TBI can undergo CT imaging as routine screening. Considering the challenges of staffing and resource limitations in parts of SA, high TBI rates and the wide spectrum traumatic events, a TBI assessment tool aimed to assist peripheral and rural emergency departments, thus allowing for standardisation of the referral and management decisions, is needed. The authors plan to study further how incorporating other criteria such as post traumatic symptoms, certain neurological signs and impaired pupillary responses to light can be considered and possibly used to generate a modified South African score, providing value to patients with TBI and healthcare personnel, thus ensuring patients that require imaging or neurosurgical referral reach tertiary centres such as IALCH in a timeous manner.

## Conclusion

As anticipated, there were significantly more CT imaging findings in the GCS 3–8 category when compared with the mild and moderate GCS categories. This finding is mainly academic as these patients are referred for further management based on established guidelines. The CT findings in patients with moderate and mild head injuries, according to the GCS, were significant and should not be missed. There were a large number of intracranial findings in a subset of patients, who may be incorrectly discharged as mild TBI. The mortality and neurosurgical intervention rate in the mild and moderate TBI categories reaffirm the need to create a standardised TBI assessment tool that can expand on the GCS. Moreover, this study showed that the GCS score alone cannot predict injury severity or mortality.

This study is part of a larger project aimed at creating a standardised TBI assessment tool assisting in training of medical personnel ensuring optimised patient management and referral. This would allow for easier discussions between referring and accepting hospitals, ensuring timeous referral of appropriate patients and preventing unnecessary imaging or patient radiation exposure and neurosurgical referrals.
